# Zero-dose children in Latin America: analysis of the problem and possible solutions

**DOI:** 10.12688/f1000research.155286.2

**Published:** 2025-01-27

**Authors:** Maria L. Avila-Aguero, Helena Brenes-Chacon, Mario Melgar, Francisco Becerra-Posada, Enrique Chacon-Cruz, Angela Gentile, Martha Ospina, Nancy Sandoval, Jennifer Sanwogou, Analia Urena, Maria T. Valenzuela, Ana Morice

**Affiliations:** 1 Latin American Society of Pediatric Infectious Diseases, San Jose, Costa Rica; 2 Affiliated Researcher, Center for Infectious Disease Modeling and Analysis (CIDMA), Yale University, New Haven, Connecticut, USA; 3 Pediatric Infectious Diseases Division, Hospital Nacional de Ninos Dr Carlos Saenz Herrera, Caja Costarricense del Seguro Social, San Jose, Costa Rica; 4 Pediatric Infectious Diseases Unit, Hospital Roosevelt de Guatemala, Guatemala City, Guatemala Department, Guatemala; 5 Global Health Department, Florida International University Robert Stempel College of Public Health and Social Work, Miami, Florida, USA; 6 CEO and funder, Think Vaccines LLC, Houston, Texas, USA; 7 Epidemiology Department, El Hospital de Ninos Ricardo Gutierrez, Buenos Aires, Argentina; 8 Advisor of Global Health Department, Florida International University Robert Stempel College of Public Health and Social Work, Miami, Florida, USA; 9 Pan American Health Organization, Washington DC, USA; 10 Centro de Estudios para la Prevencion y Control de Enfermedades Transmisibles, Universidad ISALUD, Buenos Aires, Argentina; 11 Science for Health Care Faculty, Universidad San Sebastian, Santiago, Chile; 12 International Independent consultant, San Jose, Costa Rica

**Keywords:** dose-zero children, DTP1, immunization, vaccination, National Immunization Technical Advisory Groups, NITAGs

## Abstract

**Introduction:**

Zero-dose children (ZDC) are defined as those that have never been reached by routine immunization services. In Latin America, almost 2.7 million infants younger than 1 year of age, have incomplete vaccination schedules, and vaccine preventable diseases such as measles or polio have increase worldwide. ZDC are reported to reside in high risk and fragile settings, including remote-rural areas, urban slums, and conflict-affected areas. Identifying the problem and settings in each country is mandatory to propose possible solutions to the immunization coverage situation.

**Areas covered:**

In November 2023, a group of experts of the Latin America Society of Pediatric Infectious Diseases (SLIPE) analyzed the global and regional reality of ZDC, and present in this document an updated reality of the Latin American region and the weight of the possible interventions to overcome this problem.

**Expert commentary:**

Communication is a key element to improve vaccination coverage, as it is quality and use of vaccination data. Campaigns that deliver targeted and effective messages to communities and families, provide education about vaccination, avoid missed vaccination opportunities, and coordinate efforts across different sectors and communities, among other strategies, could improve the current immunization situation.

## Article highlights

- Zero-dose children (ZDC) is a worldwide issue that could lead to reemergence of vaccine preventable diseases. Latin America is not foreign to this problem.
- The challenges of implementing strategies to address this problem is difficult, especially because there is not only one cause for this problem. Nevertheless, the measures used to this purpose could lead to adequate immunization coverage in the Latin American region.
- Communication in immunization is a key element to improve vaccination coverage, as it is quality and use of vaccination data. Campaigns that deliver targeted and effective messages to communities and families, provide education about vaccination, avoid missed vaccination opportunities, and coordinate efforts across different sectors and communities, among other strategies, could improve the current immunization situation.
- We sought to review the situation around the increase of ZDC in our region, and to present possible interventions to address this problem.

## 1. Introduction

As of January 2023, research in vaccines and vaccine development has evolved, with almost 966 new vaccine candidates. ^1^ Even though this number of these new candidates is significantly higher than 5 years ago, the use of already approved vaccines is not adequate, and the number of zero-dose children (ZDC) has also increased. ^2^ In Latin America, UNICEF reports that almost 2.7 million infants younger than 1 year of age, have incomplete vaccination schedules, and that vaccine preventable diseases such as measles or polio have increased worldwide in the last years. ^3^

The strategic plan of the Pan American Health Organization (PAHO) highlights the importance of vaccine accessibility to generate an impact and reduce disease burden. ^4^ By 2022, vaccine coverage around the world improved, and the number of children with no DTP1 dose went from 18 to 14 million children. Nevertheless, disparities among countries is evident, with just ten countries accounting for 58% of the world’s unvaccinated infants. ^5^ The World Health Organization’s (WHO) Immunization Agenda 2030 details the steps to accomplish better immunization coverage. ^6^

Latin America and the Caribbean is not foreign to this reality. In November 2023, a group of experts of the Latin American Society of Pediatric Infectious Diseases (SLIPE) analyzed the global and regional reality of ZDC. This document presents an updated reality of the Latin American region and the potential impact of various interventions to address this problem.

## 2. Current situation of zero-dose children in Latin America

ZDC are defined as those that have never been reached by routine immunization services. They are operationally measured as those who do not have their first dose of a vaccine containing diphtheria-tetanus-pertussis components (DTP1). Nevertheless, the problem does not stop with susceptible children; it also involves the lack of healthcare access for their families. This population also faces several barriers that separates them from adequate medical access, such as: socioeconomic vulnerability, inadequate access to medical services, gender, ideologic, and ethnic barriers, lack of prenatal care access, hard-to-reach communities, and regions in conflict, among others. ^7^^,^^8^ Africa, South-East Asia, and Eastern Mediterranean are among the regions with the most ZDC. Within the Americas, Mexico and Brazil were mentioned as high-risk countries during 2021 and 2022 respectively. ^7^ However, the Americas region achieved a 14% reduction in the number of children receiving zero doses from 2021 to 2022, dropping from 1.85 million to 1.31 million. ^9^

According to UNICEF estimates, by 2030 a decrease of 50% in ZDC is expected. Latin America has shown promising reduction in 2022, and a considerate reduction is possible if the problem is stipulated as a priority in the region. ^5^

In 2023, LAC documented coverage for DTP1 of 89%, higher than before the pandemic, and equivalent to more than 1 million zero-dose children in the region, distributed unevenly among countries. For example, in Venezuela, coverage with DTP1 was documented in 65% of children, while Dominican Republic, Chile, Cuba, Costa Rica, Trinidad and Tobago, Bahamas, Antigua and Barbuda, and Dominica reported coverage of 99%. Mexico, with a coverage of 89% for DTP1, was reported to be the country with highest absolute number of zero-dose children in the region, followed by Venezuela. ^10^

Measles, a highly transmissible disease, is quickly exposing any immunity gaps in a population. In LAC, coverage in 2023 was 82%, lower than the one reported before the pandemic in 2019 (86%), representing 1.6 million children with no first dose of measles vaccine in the region. In this period, nine countries documented a decrease in coverage for measles vaccine, with the largest decline documented in Mexico, from 86% in 2019, to 76% in 2023. ^10^

The global strategy of the WHO establishes a strategic objective regarding vaccination coverage based in equity, looking to achieve high vaccination coverage at national and subnational levels, especially in countries with more disadvantages. ^11^ Issues around vaccination are documented for Latin America in a PAHO evaluation of risk, ^12^^,^^13^ where they identify the implication of several diseases in the Americas, and the special situation and challenges after the COVID-19 pandemic.

Before the pandemic, cases of diphtheria were sporadically reported, mainly in Venezuela and Haiti. With vaccination coverage decrease during 2020-22, reported cases of diphtheria increased in other countries in the regions. For measles, a considerable increase of cases started to be reported in Latin American countries in 2018. With the COVID-19 pandemic and restriction measures implemented, these cases decreased, but a new increase was documented in 2023, with almost 177 suspected cases in the region. For polio, the situation is different since most reported cases in Latin America are related to vaccination. Wild polio cases primarily occur in Asian countries, but in 2019, Guatemala reported three vaccine related polio cases. Nevertheless, risk of wild polio disease based on vaccine coverage is reported, and according to the WHO, Brazil, Haiti, Dominican Republic, and Peru are considered very high-risk countries, while Argentina, Bahamas, Bolivia, Ecuador, Guatemala, Panama, Suriname, and Venezuela are classified as high-risk countries. For yellow fever, a decrease of cases has been reported by 2022. ^14^ Data of zero-dose children in several countries of Latin America is documented in Table 1.

**Table 1.** Zero-dose and under-vaccinated children in Latin America and the Caribbean in 2021.

Country	Number of zero-dose children	Number of under-vaccinated children
Brazil	709,768	163,792
Mexico	316,830	93,185
Venezuela	120,306	75,748
Argentina	112,376	37,459
Colombia	72,323	28,929
Ecuador	65,171	17,773
Haiti	64,788	62,196
Bolivia	64,400	12,880
Peru	58,837	47,069
Guatemala	40,243	36,584
Honduras	38,537	10,705
Paraguay	28,660	12,283
El Salvador	-	-
Nicaragua	16,677	1,390
Panama	5,324	14,450
Jamaica	2,298	985
Chile	2,282	9,128
Suriname	2,087	988
Dominican Republic	2,002	30,029
Uruguay	1,787	2,145
Belize	1,211	0
Cuba	1,001	0
Bahamas	971	185
Trinidad and Tobago	879	176
Costa Rica	608	0
Barbados	513	30
Grenada	413	138
Guyana	321	1,123
Saint Lucia	225	184
Antigua and Barbuda	79	11
Dominica	38	38
Saint Kitts and Nevis	17	6
Saint Vincent and the Grenadines	13	27

Source: Adapted form World Health Organization and United Nations Children’s Fund, “Estimates of National Immunization Coverage (WUENIC) 2023. ^10^

Identifying and characterizing the problem in each country is mandatory to understand the number of ZDC and undervaccinated communities, who and where they are, and why they have not been reached. Based on those answers, the country can make decisions and implement effective and sustainable interventions. There are countries where vaccines supply deficiency is identified, making the solution in these regions complex. On the other hand, there are countries where the access to population is difficult due to distrust, poverty, lower socioeconomical status, discrimination, or religious beliefs, and their approach may differ from others. ^15^

Confidence in vaccination is a determinant for vaccine acceptance, a concept severely damaged during the COVID-19 pandemic. Involvement of health care workers in shaping public perception of vaccination and education about vaccines play a major role and can help prevent vaccine hesitancy. ^16^

## 3. Risk focus evaluation of decision-making interventions for dose-zero children in Latin America

Vaccine coverage can be calculated differently in various regions. Administrative forecasts of vaccine coverage may not give a correct estimation of a country’s reality, and the real impact of vaccination may not be accurate. For example, home births in remote regions or children with an irregular migration status may not be quantified as undervaccinated or ZDC. Nominalization of immunization coverage is a strategy to geographically localize ZDC allowing for more targeted actions in affected regions. Homogeneity of vaccine coverage within each country and at regional level is also a strategy to decrease susceptible populations. For example, one of the challenges is not only to identify and vaccinate ZDC but also to keep them in the system to complete their vaccination schedules. In these cases, the “dropout” indicator, which shows how many children received DPT1 but did not receive DPT3, is crucial for monitoring if children are completing their vaccination schedule.

The lack of a unified easy-to-access system on which vaccines are updated will improve immunization report. Having an Electronic Immunization Registry (EIR) globally accessible in all countries for all vaccines and healthcare subsectors would contribute to the availability of real-time information and the development of strategies to catch up on delayed vaccination schedules and reach underserved populations. ^17^ The usefulness of a robust EIR was clearly showed during the COVID-19 pandemic when countries had to vaccinate almost their entire populations in record time. The availability of relevant, reliable, and timely information was key to designing the campaigns, generating real world evidence on vaccine effectiveness, and issuing vaccination certificates.

Active search of vaccine preventable diseases cases is an important tool to identify high-risk regions. Improvement of communication and surveillance systems could enhance national active search of reportable diseases. Effective, rapid, transparent, and clear communication and dialogue is recommended to improve trust in health systems. ^18^^,^^19^

To mitigate risk of vaccine preventable diseases, each country needs to fulfill a series of criteria such as: subnational evaluation of risk, systematic evaluation of risk contributors, mitigation plan development, including at least 80% of communities and regions of very high-risk and high-risk of disease reintroduction, and prioritize evaluation and development of risk evaluation processes. Intensive work in and with communities is necessary to avoid missed opportunities for interventions. ^13^ Interventions should not be limited to ZDC but should also include other infants at high risk due to delayed vaccine schedules. Measuring the time to complete updated immunization calendars for those younger than one year of age could be useful as an indicator.

Informed decision-making interventions are based on the best possible evidence generated from better quality tools, triangulation to improve data analysis, and use of information, considering equity, security, sustainability, and acceptability of interested parties, ^20^ while providing active work in the field to identify at-risk populations. Evidence-based decisions will contribute to prioritize interventions, evaluate situations, and implement actions. Low and middle-income countries can evaluate immunization options according to necessity and priorities using different available tools. ^21^

For example, a survey among Argentinean pediatricians evaluating vaccination practices and barriers was conducted among 1,696 responders. ^22^ Results showed that participants considered limited capacity to explain vaccination related topics to users, coupled with limited training in the topic. False contraindications of vaccinations were commonly transmitted to families, resulting in missed vaccination opportunities for children. Temporary vaccine supply issues were also reported as barriers.

## 4. Advances in National Immunization Technical Advisory Groups (NITAGs) and their role in Latin America

Immunization has been essential in preventing thousands of deaths among children in the region. The use of evidence-based information has allowed the introduction of new vaccines in immunization programs in Latin America based on local burden of disease in each country. Re-emerging diseases in the region due to different factors highlights the importance of operational NITAGs to help improve vaccination status in our countries.

In Latin America and the Caribbean, 21 out of 42 countries in the region have a NITAG, with the most recent groups formed in Haiti in 2019, Belize in 2021, and Suriname in 2022. The Caribbean region, trough the Caribbean Community, uses a unified NITAG. According to international standards, a NITAG must have an administrative or legislative base, formal Terms of References, at least five areas of expertise represented among its members (epidemiology, immunology, pediatrics, infectious diseases, and public health), have one or more meetings per year, circulated agendas, and background documents available at least one week prior to meetings, and disclosure of conflicts of interests when present. In 2022, two additional process indicators were introduced to include whether NITAGs have issued recommendations and whether any of these recommendations have been adopted by their respective health institutions and Ministries of Health. Autonomy of NITAGs should be sought trough independence from ministries of health, nevertheless, this is not always accomplished in their established structures.

During the pandemic, in 2022, measures to improve NITAG performance were developed. One of the measures included the creation of a Regional NITAG Network of the Americas (RNA), with the aim to be the main communication channel for NITAGs in the countries of the Americas; to improve the capacity of NITAGs to efficiently formulate evidence-based recommendations on immunization through collaboration; and to facilitate NITAG exchanges.

NITAGs are essential for the success of immunization programs in countries. They base their recommendations on WHO’s Strategic Advisory Groups of Experts (SAGE), and PAHO’s Regional Technical Advisory Group (TAG), as well as their own national data, among others. NITAG objectives consist in providing evidence-based recommendations on new immunization priorities, updated vaccination schedules, surveillance of vaccine preventable diseases, and the impact of immunization in different settings.

According to NITAG assessments conducted in 2020 and 2021, NITAGs in the Americas have limitations. For example, some of the advisory groups lack the presence of different experts in expected areas. In some cases, the president of the NITAG is also the representative of the Ministry of Health, making it difficult to separate both roles and creating potential conflict of interests. Also, NITAG member selection is not always uniform creating bias in opinions generated in these groups. Finally, considering that NITAG’s role is solely advisory, they face the challenge of their recommendation being rejected or simply ignored by their Ministries of Health, without the need for clear explanation for its refusal.

NITAGs have a significant role in immunization programs in the region, from experience observations, evidence-based recommendations, and valuable information that could change the development of vaccination policies in different countries. Unfortunately, several Ministries of Health do not fully value NITAG recommendations. Regarding transparency, in many countries where there is a NITAG, their recommendations are not publicly and routinely disseminated. In those cases, lack of adherence by health authorities remains hidden. In many cases, they are not perceived as essential components of immunization systems in their country. It is important to establish, reactivate, and keep NITAGs contribution in Latin America, as their participation is key in the reduction of ZDC in the Region.

## 5. Expert opinion

### 5.1 Recommendations of the Latin America Society of Pediatric Infectious Diseases (SLIPE) for the zero-dose children situation in the region

In November 2023, the expert group of SLIPE gathered in Costa Rica to analyze the regional situation of ZDC. A series of situations that could impact immunization coverage in the region were identified and are presented in this section.

Efficiency of vaccination systems in Latin America is influenced by different situations that can have an impact in social and communal behavior towards vaccination. ^8^ Adapted and sustainable measures to overcome barriers presented in vaccination practices can be approached using integrative strategies in health systems among different countries. Identify, reach, monitor, measure, and advocate should be used as steps to analyze the situation of ZDC.

Vaccination coverage improvement objectives are not targeted to avoid reintroduction of vaccine preventable diseases in the region, -a very difficult task-, but to overcome the number of ZDC and undervaccinated communities in the region and prevent disease in vaccinated individuals and communities through herd protection of vaccine preventable diseases.

Irregular migration is a reality in the Americas and countries should adapt to these situations. For example, among the proposed strategies, the development of a Pan-American unique immunization card could be considered to give continuity to immunization schedules of migrants regardless of the country.

Latin American countries should move from evaluation of the situation only, to the implementation of strategies and communication of those successful measures with other countries. This could be accomplished by taking a broader look at the reality of the Latin American situation.

The role of NITAGs in this path is crucial. Besides the technical role of this advisory group, they are referral groups that review the global situation analysis performed yearly by the WHO and UNICEF. Data on vaccine preventable diseases are reported by country each year to the joint reporting form, providing a wide analysis for NITAGs to consider. ^23^ Recommendations provided by NITAGs should be carefully reviewed by their Ministries of Health. In the event that the Ministry of Health refuses a NITAG recommendation, a clear explanation for its refusal should be provided in writing. The NITAG should then be able to identify a new policy question to address the issue and follow up with the Ministry of Health for further discussion. A broader understanding of epidemiological realities in every country is necessary for the success of implemented strategies to decrease the number of ZDC in our region.

To reduce the number of ZDC and unvaccionated communities in Latin America, a comprehensive strategy should be implemented. Here are some key components of such a strategy:

1. Improve vaccine accessibility ensuring vaccines are readily available and accessible to all children, even in remote or underserved areas. This can be achieved through mobile vaccination units, extended clinic hours, or outreach programs to reach marginalized populations.
2. Increase awareness, education and collaboration with communities and stakeholders to implement awareness campaigns to educate parents, caregivers, and communities about the importance of vaccines in preventing diseases. Education should start early in school programs using awareness activities among children and families. Also, incorporating education regarding vaccines and their policies during medical education could improve caregivers’ knowledge. Address myths and misinformation about vaccines through evidence-based information campaigns could help engage with community leaders, religious groups, healthcare providers, and other stakeholders to build trust in vaccination programs. Foster partnerships to address cultural, social, and economic barriers to vaccine uptake.
3. Robust monitoring and evaluation of vaccination coverage to track vaccination coverage rates at national, regional, and local levels. Use data to identify underserved areas and populations in need of targeted interventions. Adopt relevant recommendations to support, strengthen, report, and monitor vaccine preventable diseases in each country, could improve quality and use of data. ^24^ Standard coverage estimates are based on the distributive-administrative method, which is practical, nonetheless, it does not give information related to incomplete courses neither on vaccine recipients, and it relies heavily on quality of reporting. ^25^
4. Address equity and inequalities ensuring that vaccination programs are equitable and reach all children, regardless of their socio-economic status, ethnicity, or geographic location. Target resources towards vulnerable populations to bridge existing gaps in access. Modernize vaccination schedules, by offering similar vaccines in both the public and private sectors, the use of combined vaccines, such as pentavalent or hexavalent vaccines, has been shown to be safe, effective, and well-received by the population. Their use would help prevent missed vaccination opportunities.
5. Improve vaccination registries in the region. Implementing an electronic vaccination registry could ensure accurate tracking of immunization data, optimizing vaccine distribution, enhancing public health monitoring, and ultimately improving the region’s overall health outcomes by facilitating timely and effective vaccination strategies. Although there are validated technologies to implement electronic registry of health, they have not been validated in newborns and children. ^26^ The Pan American Health Organization have been developing a methodology to assess electronic immunization registries in low- and middle-income countries in Latin America to evaluate health information systems, framework, and strategies to assess information systems. ^27^ Moving towards EIR should be a priority for countries that have not implemented this tool yet.

A recent publication analyzed the insights to support the planning, development, and implementation of vaccination in several countries in the LAC region, to provide an equity-focused COVID-19 vaccination policies for the region. They identified the need of multi-level and intersectoral alliances to improve vaccination coverage, looking for collaboration among education entities, indigenous affairs, civil and social organizations, and leaders in the community among other to promote immunization in all sectors of society. ^11^

Our study has limitations. Reliable data on vaccine coverage is not consistently available across all regions of Latin America, and inferences often rely on reports submitted to international entities, which may introduce bias. Additionally, the incidence of zero-dose children could be under- or overestimated in these global analyses. Future studies require specific and reliable data to address these gaps effectively.

## 6. Conclusions

ZDC is a worldwide situation that could result in a reemergence of vaccine preventable diseases. Latin America and the Caribbean are not exempt of this reality, and the associated health impact makes it a priority concern. Understanding the challenges around ZDC, the reality and specific needs of every country region and implementing strategies to address this problem are the next steps in reaching adequate immunization coverage.

Communication in immunization is a key element to improve vaccination coverage, as it is quality and use of vaccination data. Campaigns that deliver targeted and effective messages to communities and families, provide education about vaccination, avoid missed vaccination opportunities, and coordinate efforts across different sectors and communities, among other strategies, could improve the current immunization situation.

Addressing the challenge of zero-dose children in Latin America requires unwavering political will, as it is the cornerstone for mobilizing resources, strengthening health systems, and fostering equitable access to immunization services.

The field of vaccine development has advanced in the last years, including research for better antigenic affinity, broader antigenic efficacy and effectiveness, easier and faster manufacturing, and lower costs. However, vaccine technology must be integrated with comprehensive public health policies to reduce the number of ZDC in Latin America. This integration should encompass not only the development and distribution of vaccines but also the implementation of educational programs, community outreach, and robust healthcare infrastructure. By ensuring that technological advancements in vaccination are supported by strong policy frameworks, we can enhance access to vaccines, improve immunization rates, and ultimately protect more children from preventable diseases.

## Author contributions

All authors contributed equally to the conception, design, information, manuscript preparation, and edition.

## Data Availability

No data are associated with this article.
