# Supplementary material for: Zero-dose children in Latin America: analysis of the problem and possible solutions
Source: F1000Res. 2025 Jan 27;13:1060. Originally published 2024 Sep 17. [Version 2] doi: 10.12688/f1000research.155286.2 (PMC11966093; doi:10.12688/f1000research.155286.2)
Supplement: Peer review history

# Reviewer report: Reviewer response for version 2

**DOI:** 10.5256/f1000research.175250.r371896

**Published:** 2025-04-02

**Authors:** Yangyupei Yang

**Affiliations:** 1 Johns Hopkins Bloomberg School of Public Health, Baltimore, USA

This opinion article titled *“Zero-dose children in Latin America: analysis of the problem and possible solutions”* by Avila-Aguero et al. addresses the critical issue of children in Latin America who have never received a routine vaccine dose — commonly referred to as “zero-dose children” (ZDC). The article synthesizes available data and insights from regional experts to examine the magnitude, geographic distribution, and underlying causes of this problem. It also outlines recommended interventions, including health system improvements, education and outreach strategies, and the strengthening of National Immunization Technical Advisory Groups (NITAGs). The paper emphasizes that political will and intersectoral collaboration are essential to achieving immunization equity in the region. This article is a valuable expert commentary that addresses a pressing public health issue in Latin America. With minor revisions to improve the clarity of evidence, and regional contextualization, it will serve as an insightful resource for both academic and policy audiences. I recommend acceptance with minor revisions.

**Minor comments:**

 ·  There are several minor grammar or phrasing issues. A final round of language polishing is recommended.

 ·  Consider defining abbreviations (e.g., DTP1, EIR, SLIPE) at first mention for accessibility to broader audiences.

 ·  Citations for some factual claims (e.g., vaccine hesitancy or COVID-19’s impact on ZDC) could be strengthened.

Is the topic of the opinion article discussed accurately in the context of the current literature?

Yes

Are arguments sufficiently supported by evidence from the published literature?

Yes

Are all factual statements correct and adequately supported by citations?

Yes

Are the conclusions drawn balanced and justified on the basis of the presented arguments?

Yes

Reviewer Expertise:

Immunization, global health, zero dose

I confirm that I have read this submission and believe that I have an appropriate level of expertise to confirm that it is of an acceptable scientific standard.

# Reviewer report: Reviewer response for version 2

**DOI:** 10.5256/f1000research.175250.r362833

**Published:** 2025-02-11

**Authors:** Anne Eudes Jean Baptiste

**Affiliations:** 1 World Health Organization, Abuja, Nigeria

Please include the data source in the table.

 No further comments – Approved from my side.

Is the topic of the opinion article discussed accurately in the context of the current literature?

Yes

Are arguments sufficiently supported by evidence from the published literature?

Yes

Are all factual statements correct and adequately supported by citations?

Partly

Are the conclusions drawn balanced and justified on the basis of the presented arguments?

Partly

Reviewer Expertise:

Immunization, epidemiology, surveillance, measles, polio, diphtheria, tetanus, pertussis and zero-doses.

I confirm that I have read this submission and believe that I have an appropriate level of expertise to confirm that it is of an acceptable scientific standard.

# Reviewer report: Reviewer response for version 1

**DOI:** 10.5256/f1000research.170438.r329401

**Published:** 2024-10-10

**Authors:** Chelsea Marie

**Affiliations:** 1 Division of Infectious Diseases and International Health, University of Virginia School of Medicine, Charlottesville, VA, 22908, USA

This review entitled "Zero-dose children in Latin America: analysis of the problem and possible solutions" seeks to describe the problem of Zero Dose Children (defined as those that have never been reached by routine immunization services), describe how interventions can be targeted, and suggest solutions. The review also includes a discussion on the importance and role of National Immunization Technical Advisory Groups (NITAGs).

 The topic is highly significant as ZDC are vulnerable to preventable diseases and the number of ZDC in a country is a key indicator of barriers to vaccination. Analysis of vaccination is particularly topical in the wake of the covid-19 pandemic which resulted in structural barriers to vaccination as well as an increase in vaccine hesitancy in some populations. 

 While the title suggests an analysis of ZDC in Latin America the content presented herein is more editorial in nature, with a mixture of global, regional and country-wide statistics presented throughout that while illustrative of some specific points, do not leave the reader with a comprehensive understanding of the problem or it's associated impacts. 

 If within the scope, I believe this review has the unique opportunity to include such an analysis by including an analysis of ZDC in Latin America in a country by country manner from the WHO and UNICEF Estimates of National Immunization Coverage (WUENIC), 2023 revision report. . Approaching the evaluation of ZDC in Latin America in the way could highlight not only the countries where interventions are most needed, but also highlight countries that have effectively reduced the proportion of ZDC which could in turn suggest or inform the section on possible solutions. While the estimates in this report come with limitations, the authors appear to be well-qualified to offer an expert interpretation and discussion of such limitations. 

 The prevalence of vaccine preventable diseases in Latin America is mentioned in several places throughout the review and similar to the comment above, including a comprehensive analysis of prevalence estimates of vaccine preventable diseases would strengthen this review and offers the opportunity to examine how ZDC rates and vaccine preventable disease rates intersect. 

 In the section entitled "Risk focus evaluation of decision-making interventions for dose-zero children in Latin America". I believe the goal of this section is to summarize how data can be used to prioritize and target interventions for ZDC across Latin America. I agree that the discussion of drop-out is very important and a key factor beyond ZDC that should be included. This could be analyzed by looking at the rates DPT1 and DPT3 by country in the WUENIC report and would be a fascinating adjunct that has the potential to highlight distinct barriers by country. 

 There is a brief mention of the impact of the covid-19 pandemic on vaccination rates, that is also apparent in the data on vaccine rates. It would be interesting to discuss the impact in terms the decrease in vaccination rates during the pandemic, as well as the rate of catch-up by country. The important topic of vaccine hesitancy is also mentioned and is interesting as it contradicts the assumption that ZDC is largely driven by lack of access. Is vaccine hesitancy thought to be a driver of ZDC in Latin America? 

 The authors highlight the key role of Advances in National Immunization Technical Advisory Groups (NITAGs) in Latin America. As well as barriers faced by NITAGs. This is an important topic and offers useful information. This section could be strengthened by including more detailed information on the countries with and without NITAGs, as well as include references for some of the statements in this section. 

 The final section highlights broad recommendations of the SLIPE  to improve immunization rates and decrease the number of ZDC in Latin America. These recommendations are fairly common-sense and apply to vaccination efforts throughout the world. This impact of this section could be increased by placing these recommendations in the context of the existing landscape of ZDC in Latin America  and highlighting region specific or country specific barriers. 

 Overall, this review offers information on a crucial public health issue of ZDC in Latin America. My recommendations for approval would be to include a more comprehensive analysis of the problem leveraging the UNCEIF and WHO data and to provide more focused  recommendations on the particular barriers facing Latin American countries.

Is the topic of the opinion article discussed accurately in the context of the current literature?

Partly

Are arguments sufficiently supported by evidence from the published literature?

Partly

Are all factual statements correct and adequately supported by citations?

No

Are the conclusions drawn balanced and justified on the basis of the presented arguments?

Partly

Reviewer Expertise:

Microbiology, immunology and clinical research.

I confirm that I have read this submission and believe that I have an appropriate level of expertise to confirm that it is of an acceptable scientific standard, however I have significant reservations, as outlined above.

# Response

**Published:** 2024-11-24

**Authors:** Helena Brenes

**Affiliations:** Pediatric Infectious Diseases, Not Applicable, San Jose, Costa Rica

Thank you for the observations. We appreciate every comment that was done to improve our manuscript. The objective of this article was not to analyze the reality in every country of the region, but to analyze the general problem and give general possible solutions to this issue. We emphasize our results in the available information and included more data of the countries that participated in this review. Changes are highlighted in red.

# Reviewer report: Reviewer response for version 1

**DOI:** 10.5256/f1000research.170438.r325745

**Published:** 2024-10-01

**Authors:** Anne Eudes Jean Baptiste

**Affiliations:** 1 World Health Organization, Abuja, Nigeria

To strengthen the article, several key areas require attention:

 1- More quantitative data on immunization coverage rates, ZDC prevalence, and relevant metrics would enhance the analysis and identification of specific areas of concern.

 2- Acknowledging limitations, such as potential bias in data analysis, and outlining areas for future research is essential. This will enhance the article's credibility.

 3- Emphasizing in the conclusion the importance of political will and commitment is critical.

Is the topic of the opinion article discussed accurately in the context of the current literature?

Yes

Are arguments sufficiently supported by evidence from the published literature?

Yes

Are all factual statements correct and adequately supported by citations?

Partly

Are the conclusions drawn balanced and justified on the basis of the presented arguments?

Partly

Reviewer Expertise:

Immunization, epidemiology, surveillance, measles, polio, diphtheria, tetanus, pertussis and zero-doses.

I confirm that I have read this submission and believe that I have an appropriate level of expertise to confirm that it is of an acceptable scientific standard, however I have significant reservations, as outlined above.

# Response

**Published:** 2024-11-24

**Authors:** Helena Brenes

**Affiliations:** Pediatric Infectious Diseases, Not Applicable, San Jose, Costa Rica

Thank you for the observations. We appreciate every comment that was done ot improve our manuscript. The objective of this article was not to analyze the reality in every country of the region, but to analyze the general problem and give general possible solutions to this issue. We emphasize our results in the available information and included more data of the countries that participated in this review. Changes are highlighted in red.

# Response

**Published:** 2024-11-24

**Authors:** Helena Brenes

**Affiliations:** Pediatric Infectious Diseases, Not Applicable, San Jose, Costa Rica

**Reviewer Comments:**

 1. More quantitative data on immunization coverage rates, ZDC prevalence, and relevant metrics would enhance the analysis and identification of specific areas of concern.

***Author Response: *** We added available information of the regions where information is available, and including countries that participate in this review.

 2. Acknowledging limitations, such as potential bias in data analysis, and outlining areas for future research is essential. This will enhance the article's credibility.

***Author Response: *** Thank you, we added a limitation section to clarify this aspects.

 3. Emphasizing in the conclusion the importance of political will and commitment is critical.

***Author Response: *** Thank you, we added a sentence with this comment at the conclusion section.
